# Role of Thermal Ablation in Colorectal Cancer Lung Metastases

**DOI:** 10.3390/cancers13040908

**Published:** 2021-02-22

**Authors:** Alexandre Delpla, Thierry de Baere, Eloi Varin, Frederic Deschamps, Charles Roux, Lambros Tselikas

**Affiliations:** Interventional Radiology Unit, Medical Imaging Department, Gustave Roussy, 94800 Villejuif, France; thierry.debaere@gustaveroussy.fr (T.d.B.); eloi.varin@gustaveroussy.fr (E.V.); Frederic.DESCHAMPS@gustaveroussy.fr (F.D.); charles.roux@gustaveroussy.fr (C.R.); lambros.tselikas@gustaveroussy.fr (L.T.)

**Keywords:** ablation, metastases, colorectal, lung

## Abstract

**Simple Summary:**

For a long time, surgery has been the only local treatment for pulmonary metastases. Percutaneous thermal ablation appeared in the early 2000s as a minimally invasive alternative technique to surgery for patients who were not eligible for surgery or wanted to preserve quality of life. In this review, we discuss the role of thermal ablation in the management of lung metastases of colorectal cancer, and present the main results of the literature concerning oncological outcomes (local tumor control, survival) based on 12 relevant original studies each involving a minimum of 50 patients, with a minimal follow-up of 12 months.

**Abstract:**

*Background*: Consensus guidelines of the European Society for Medical Oncology (ESMO) (2016) provided recommendations for the management of lung metastases. Thermal ablation appears as a tool in the management of these secondary pulmonary lesions, in the same manner as surgical resection or stereotactic ablative radiotherapy (SABR). *Methods*: Indications, technical considerations, oncological outcomes such as survival (OS) or local control (LC), prognostic factors and complications of thermal ablation in colorectal cancer lung metastases were reviewed and put into perspective with results of surgery and SABR. *Results*: LC rates varied from 62 to 91%, with size of the metastasis (<2 cm), proximity to the bronchi or vessels, and size of ablation margins (>5 mm) as predictive factors of LC. Median OS varied between 33 and 68 months. Pulmonary free disease interval <12 months, positive carcinoembryonic antigen, absence of neoadjuvant chemotherapy and uncontrolled extra-pulmonary metastases were poor prognostic factors for OS. While chest drainage for less than 48 h was required in 13 to 47% of treatments, major complications were rare. *Conclusions*: Thermal ablation of a selected subpopulation of patients with colorectal cancer lung metastases is safe and can provide excellent LC and delay systemic chemotherapy.

## 1. Introduction

Colorectal cancer is the second most commonly diagnosed cancer worldwide. Twenty percent of patients have metastases at diagnosis [1]. The lung is the second most common site of colorectal cancer metastasis after liver. During the course of the disease, approximately 20% of patients will develop one or more pulmonary metastases [2,3].

Until a few years ago, metastatic cancer was considered the end stage of the disease. Today, efforts are directed towards improving the prognosis of these patients through a multi-modal and multi-disciplinary approach.

Although the overall survival benefit of local treatment of colorectal cancer pulmonary metastases has never been proven in randomized studies, complete cure can be obtained in some patients, namely in oligometastatic patients. For a long time, surgery has been the standard and only treatment for these pulmonary metastases, and some benefit for survival seems to be demonstrated in large non randomized studies [4,5]. Many patients are ineligible for thoracic surgery because of their comorbidities, even more so for repeated lung metastatsectomy, namely because resection can be the cause of deterioration in respiratory function [6]. Percutaneous ablation appeared in the late early 2000s as a minimally invasive alternative technique to surgery for patients who were not eligible for surgery or wanted to preserve quality of life.

In this review, we discuss the role of thermal ablation in the management of pulmonary metastases of colorectal cancer, and present the main results of the literature for oncological outcomes (local tumor control, survival) based on 12 relevant original studies involving a minimum of 50 patients, with a minimal follow-up of 12 months.

## 2. Indications

Management of colorectal cancer lung metastases requires a decision to be taken by a multidisciplinary team (MDT), based on local experience, tumor characteristics and patient preference. The ESMO consensus guidelines for the management of patients with metastatic colorectal cancer provides some specific recommendations for the management of lung metastases [7]. In these guidelines, local ablative therapies incorporating surgery, thermal ablation, and stereotactic ablative radiotherapy (SABR) are recommended when suitable, in combination with systemic therapies, and especially in an oligometastatic population.

Thermal ablation of lung metastases, aims at complete disease control or non-evaluable disease in the same manner as surgical resection, consequently indications encompass a controlled primary tumor, an exclusive pulmonary metastatic disease (except for curable distant metastases) and a technically feasible “R0” lung ablation. Untreatable coagulation disorders are the only absolute contraindications to pulmonary thermo-ablation. However, it is possible to define patients who do not appear to be good candidates for lung ablation, as described in CIRSE standards of practice guidelines [8]: patients with an Eastern Cooperative Oncology Group performance status of 2 or more and a life expectancy of less than 1 year. Patients with more than five lung metastases or a metastasis with a maximum diameter >30 mm must be thoroughly discussed to weigh the benefit and risk of such treatment.

Required pre-treatment imaging includes a chest CT scan that is less than 4 weeks old to determine patient eligibility. In practice, a chest CT scan may be performed before general anesthesia and the procedure [9]. PET-CT could be used to rule out possible extra-pulmonary locations of disease. A relevant clinical history with concordant imaging of lung nodules may sufficiently support an MDT decision to proceed with treatment. However, when the nodule is unique, whether it is diagnosed synchronously with the primitive cancer or, on the contrary, largely at a distance from the remission, the need for a histological diagnosis arises. A study involving the retrospective analysis of the histology of 140 solitary nodules surgically resected in cancer patients showed that only 50% of the lesions were actually metastases, 26% were primary cancers and 24% were benign lesions [10]. We then understand the necessity, in unclear cases, to perform a biopsy in order to decide in particular on the continuation of the oncological management (perioperative chemotherapy). The biopsy is best performed during the same procedure as the ablation, to avoid further hospitalization or complications such as pneumothorax. Performing a biopsy just before the ablation, during the same procedure, may compromise the technical success of the ablation. Indeed, the occurrence of a pneumothorax or alveolar hemorrhage may make it difficult to target the lesion to be treated. The technique, which consists of placing the treatment needle and then performing the biopsy through another coaxial system, requires a second puncture and therefore may increase the risk of complications. Finally, it has been shown that the histological diagnosis of malignancy can be made in 90% of cases from a biopsy immediately performed after lung radiofrequency ablation (and 70% for the cancer subtype) [11]. This trick has the advantage of not compromising the success of the thermal ablation.

## 3. Techniques

The lung is percutaneous thermo-ablation friendly both for image guided tumor targeting and thermal heating delivery. For image guidance under CT or CBCT imaging [12], the low aeric density of the normal lung parenchyma and metastatic tissue density provides excellent visualization of the needle for navigation and can evaluate accurate targeting of the lesion to be treated. For heat delivery, the healthy lung around the metastasis, thanks to the thermal and electric insulation of air, limits energy loss and allows concentration of energy in the target tumor as well as larger ablation sizes then that obtained in other soft tissues such as the kidney or liver [13]. 

Among the three main techniques of thermal ablation including radiofrequency, microwave and cryotherapy, radiofrequency is the most reported technique over the past 20 years [14]. Radiofrequency requires grounding pads and is limited to a single applicator that can be fired at the same time. A target temperature of 60 °C at the tip of the electrode causes cellular death [15]. Size and shape of the ablation zone depends on the material used, the power and the heating time. Blood vessels cool tissues in contact and reduce the size of the ablation zone, because of the heat sink due to convective cooling by vessels [16].

The microwave, based on the heating of the water molecules within the tissue, makes it possible to overcome a conductivity defect in the tissue surrounding the electrode. It thus allows larger ablation volumes in a shorter period of time with a single needle. It has the advantage of allowing for firing of several needles at the same time. Its main disadvantages are slightly higher cost and some lack in reproducibility of the ablation volume for a given algorithm of treatment, as well as the elongated shape of the ablation volume. A study on the microwave ablation of 43 porcine lung tumor mimics reported a coefficient of variation of 16 to 30% in continuous mode and 20 to 46% in pulsed mode. The reproducibility rate was moderate [17]. The sphericity index is poor regardless of the ablation system used or the manufacturers’ algorithms [18]. In clinical practice, microwave seems to be less impacted by the heat sink effect and vessel proximity [19].

Microwave and radiofrequency can be performed under conscious sedation, but proximity of the pleura may cause pain during treatment. In addition, the use of sedation may be difficult with patients who cannot cooperate properly or cannot hold their breath. Finally, many teams will prefer general anesthesia because the anesthetist can take care of the patient and monitor vital signs, while the radiologist can focus on the therapeutic procedure. A retrospective study comparing feasibility, complication rate and local tumor control after pulmonary tumor radiofrequency ablation did not show a difference between conscious analog-sedation and general anesthesia [20].

Cryotherapy is based on the cooling of tissues, inducing cell death from −20 °C. Most of the time, several cryoprobes are necessary to cover supra-centimetric lesions and the unit cost of these is high [21], but cryotherapy, unlike RFA, is not limited by the number of probes that can be activated at the same time. The use of the “stick mode” makes it possible to move the targeted lesion away from a risky structure (using freezing at reduced power to stick the tissue around the cryoprobe) and thus if needed to insert a second needle with precision, taking advantage of the fact that the target metastasis can be held in place by the first probe. Thanks to the low incidence of per-procedural pain during cryotherapy, it can be performed under conscious sedation and/or local anesthesia. This has the advantage of avoiding intubation, which is conducive to the formation of atelectasis. These can be responsible for difficulties in targeting small peripheral lesions. The standard protocol for lung cryotherapy includes a short cooling cycle at the beginning of the treatment, allowing an inflow of liquid into the pulmonary alveoli. This facilitates ice ball formation and good visualization of the treatment zone. The ice ball can be monitored in real time under computed CT guidance [22].

## 4. Oncological Outcomes

We conducted a review of the current literature, following PRISMA guidelines, to describe local tumor control, survival and adverse events after thermal ablation of pulmonary metastases of colorectal cancer.

**Figure 1:**
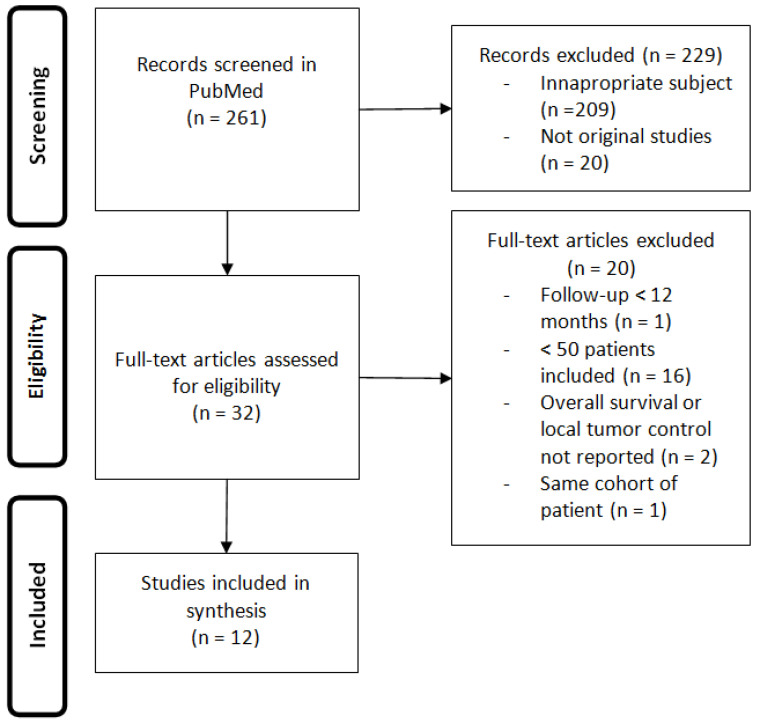
Consort diagram.

The search items in PubMed were ((colorectal AND (lung OR pulmonary) AND (metastases OR metastasis) AND (ablation OR radiofrequency OR cryoablation)). We included human original studies in English, in patients with colorectal cancer lung metastases treated with thermal ablation. We excluded studies involving less than 50 patients, studies with a follow-up less than 12 months, studies that do not report overall survival or local tumor control. Full articles were analyzed and reference lists were screened for additional material. The searches were conducted on 10 February 2021 (Figure 1), and revealed 261 articles from Pubmed. A total of 32 articles were eligible and 20 did not meet the inclusion criteria.

Results of the 12 relevant original studies involving more than 50 patients treated with thermal ablation in the management of pulmonary metastases of colorectal cancer, with a minimal follow-up of 12 months, are reported in Table 1.

### 4.1. Local Control

The reported local control (LC) rate, defined by the absence of recurrence at the ablation site, varied from 62 to 91%. The local control rate improved in the most recent studies, probably due both to technical improvement and patient/tumor selection including knowledge of risk factors for local tumor recurrence. This local control appears to persist over the years of follow-up, with a rate of up to 91% at 3 years in small size tumors [23].

Repeat ablation can improve the overall local control [24]. Indeed, in a series of 1037 lung metastases treated with radiofrequency ablation, including 52% of colorectal origin, 37 local tumor progressions were retreated with RFA. The rates of patients with locally untreatable lung progression at 1 and 2 years were, respectively, 27.6% and 38.3%. The overall survival rates of the retreated patients were, respectively, 89.8% and 82.0%, 2 and 3 years after the first pulmonary progression retreated by RFA [25]. In a series of 114 patients with 202 metastases treated with cryoablation, among the 25 patients that had local recurrence with 30 tumors, 11 patients with 12 tumors were retreated with CA, thus achieving an overall local tumor efficacy including these patients with retreatment, resulting in secondary local recurrence-free response in 184 of 202 (91.1%) [26]. In a consecutive series of 209 patients with CRC metastases, 64 out of the 126 patients presenting with lung progression were treated with TA again [27].

Surgical lung metastasectomy obtains a R0 resection roughly 90% of the time [28]. SABR has been reported in a recent meta-analysis with LC rates at 1, 2 and 3 years estimated to be 81%, 66% and 60% [29], but the LC rate was significantly lower for colorectal cancer lung metastases compared to non-colorectal cancer lung metastases (HR, 2.93; 95% confidence interval, 1.93–4.45; *p* < 0.000001).

### 4.2. Survival

Median overall survival (OS) after thermal ablation of lung metastases varied between 33 [30] and 68 months [27], with differences probably explained by heterogeneity of patient enrolled in the studies. Indeed, some studies enrolled only patients considered as nonsurgical candidates with a minimum of 4 metastases, and multilobar metastases, bilateral disease, or poor performance status [30], while others treated more than 70% of patients with one or two lung metastases [27]. The 5 year-OS consequently varied from 20 to 61% [31,32]. The OS of patients treated with RFA seems comparable to what was reported in recent reports of surgical resection, with an overall survival at 5 years ranging from 32 to 65% [33,34,35] and a median overall survival of 35 to 70 months. As of today, SABR seems to provide lower OS, of around 52% at 3 years in a large meta-analysis [29].

The reported median disease free survival (DFS) is around 8 months (63.9% at 6 months, 33.1% at 1 year, 18.4% at 2 years and 11.2% at 5 years) [27,32]. Early detection of new pulmonary metastases in patients under strict follow-up with CT scans makes it possible to treat these new metastases with a new session of thermal ablation, when needed. It is noteworthy that at the time of lung progression, 90% of patients had lung-only disease [32]. Repeated ablation of new distant lung metastases was used in 99 metastases in a series of 566 patients with 1037 metastases previously treated with RFA [24]. In a series of 114 patients with 202 metastases, among 30 patients who had recurrence with 41 distant tumors, 13 patients with 16 tumors were retreated with CA [26].

**Table 1:** Results from 12 relevant original studies involving more than 50 patients treated with thermal ablation in the management of pulmonary metastases of colorectal cancer, with a minimal follow-up of 12 months.

Study	Type of Study	Technique	Number of Patients	Inclusion Dates	Mean Size of Lesions (cm)	Median Follow Up (Months)	LTC	LTP at 1 Year	LTP at 3 Years	Median OS (Months)	OS at 1 Year	OS at 3 Years	OS at 5 Years
T.D. Yan (2007) [30]	Retrospective	RFA	55	-	2.1 +/− 1.1	24	62%	-	-	33	85%	46%	-
K. Yamakado (2009) [36]	Retrospective	RFA	78	2002–2008	2.0 +/− 1.0	24	86%	10%	21%	38	84%	56%	35%
T.C. Chua (2010) [37]	Retrospective	RFA	100	2000–2010	-	23	-	-	-	36	87%	50%	30%
A. Gillams (2013) [38]	Retrospective	RFA	122	2002–2011	1.7 (0.5–4)	12	-	-	-	41	-	57%	-
T. de Baere (2015) [25]	Retrospective	RFA	566 (293 mCCR)	2002–2010	1.7 +/− 0.9	36	92%	10%	18%	62	93%	76%	56%
J. Ferguson (2015) [31]	Retrospective	RFA	157	2000–2013	1.6 +/− 0.6	28	88%	-	-	33	89%	44%	20%
Y. Matsui (2015) [39]	Retrospective	RFA	84	2001–2012	1.5 +/− 0.7	38	86%	12%	18%	67	95%	65%	52%
I. Kurilova (2018) [32]	Retrospective	MWA	50	2011–2016	1.0 (0.3–3.2)	26	90%	7%	14%	59	94%	82%	61%
M. Fonck (2018) [27]	Retrospective	RFA, MWA, CA	209	2002–2013	1.0 (0.2–4.6)	50	-	-	-	68	95%	-	55%
J. Zhong (2019) [40]	Retrospective	RFA	70	2008–2014	1.4 +/− 0.6	46	90%	3%	10%	52	97%	75%	44%
M.R. Callstrom (2020) [26]	Prospective	CA	128 (63 mCCR)	2014–2016	1.0 +/− 0.6		91%	9%	16%	-	98%	-	-
T. Hasegawa (2020) [23]	Prospective	RFA	70	2008–2014	1.0 +/− 0.5	57	91%	-	9%	-	-	84%	-

RFA: radiofrequency, MWA: microwave, LTC: local tumor control, LTP: local tumor progression, OS: overall survival, mCCR: lung metastases of colorectal cancer, CA: cryoablation.

Other interesting parameters to evaluate when discussing local treatment of cancers are the chemotherapy-free-survival (CFS) and quality of life. CFS probably improves quality of life by postponing the use of systemic treatment. Moreover, it can save systemic therapy for more advance stages, while efficacy of chemotherapy is known to exhaust with time. Therefore, local treatment can be seen as a line of treatment. In a consecutive series of 209 patients that underwent 323 TA procedures for 630 lung metastases, the median CFS was 12.2 months (95% CI: 10.3–17.7). Patients with no extra-pulmonary metastases showed a statistically better CFS than those who had extra-pulmonary metastases with a median of 20.9 and 9.2 months, respectively (*p* < 0.001) [27]. Few data exist on quality of life for thermal ablation of lung metastases. For colorectal liver metastases, a prospective study that evaluated two comparable populations treated either with local ablative therapy or chemotherapy showed that quality of life was better in the ablative therapy group with a QUALY of 317 and 165 respectively, while median OS was not different at 31 months and 26 months, respectively [41].

### 4.3. Prognostic Factors

#### 4.3.1. Local Control Factors

The two most often reported factors of treated lesions that are prognostic for local control after thermal ablation, include a size up to 20 mm [36,42] and a number of lesions up to 2 [42]. These are not a strict obstacle to treatment of larger or more numerous lesions, provided that the ablation can be complete (R0) with minimal safety margins of 5 mm, and that all pulmonary metastases are treated, even if it requires several subsequent ablation sessions. The environment in contact with the treated lesion has been reported to impact on local control, including contact of the targeted metastasis with a vessel larger than 3 mm in diameter due to the possible heat sink effect [43], central location of the lesion, probably by increasing the vascular density near the hilum [44], or a bronchi, likely also due to the heat sink effect [24]. Finally, local control depends on the ablation margins with better outcomes for ablation margins >5 mm [45]. It is mandatory to evaluate ablation margin during the procedure, in order to provide a new location of treatment, if needed. Due to thermally induced ground glass opacity, repeated small CT scan volumes centered on the lesion allows nearly real time multiplanar evaluation of these margins with limited X-ray exposure of the patient.

#### 4.3.2. Survival Factors

Knowledge of factors associated with decrease in survival allows a better selection of patients to be treated. The early onset of pulmonary metastases of colorectal cancer appears to be an independent factor in poor survival prognosis. For Chua and Cheng, this pulmonary free disease interval is a negative prognostic factor when inferior to 12 months [37,42]. Carcinoembryonic antigen (CEA) is a biomarker used in the monitoring of patients with colorectal cancer. A normal value of CEA prior to thermal ablation is described as a predictor of prolonged survival [23,36]. However, there is no consensus on the cut-off value. For many authors, the absence of neoadjuvant chemotherapy is an independent factor in decreasing survival [23,36,37]. The value of peri-operative chemotherapy for pulmonary metastases is not specifically established in the ESMO recommendations. Nevertheless, if we compare with liver metastases of colorectal cancer, adjuvant chemotherapy of 3 to 6 months duration is recommended. Presence of extra-pulmonary metastases [36,37] appears to be a poor prognostic factor and even a possible contraindication to local treatment of pulmonary metastases, especially when other localizations are not technically feasible with the objective of curative treatment. Intensive neo-adjuvant chemotherapy must then be performed in order to re-discuss the indication for ablation in the case of a good response. Size and number of metastases have been clearly highlighted as prognostic factors for local control and remain prognostic for survival. Indeed, a median survival of 51 months, with a 3-year OS of 64% have been reported for tumors 2 cm or smaller vs. 31 months and 44% for tumors 2.1–4 cm (*p* = 0.08) [38]. In a population of 293 patients with colorectal cancer metastases, size >2 cm (HR = 2.10, *p* = 0.0027) and number of metastases ≥3 (HR = 1.86, *p* = 0.011) were significantly associated with both local control and OS [25]. 

#### 4.3.3. Adverse Events

Preserving quality of life through a low morbidity rate is a major strength of pulmonary thermal ablation and part of tumor board choice at the time of treatment allocation. The results of the 12 previously analyzed studies are reported in Table 2. The most common adverse event occurring during lung thermal ablation is pneumothorax, which requires drainage in 13 to 47% [27,36] of treatments. Most chest tube drainages are for 24 to 48 h, and when compared to surgery where the chest tubes are larger and more prolonged, such small size and short duration chest tubes must be questioned to be qualified as adverse events or part of the treatment. Other most frequently reported adverse events include pleural effusion, pneumonia or asymptomatic alveolar hemorrhage. Major complications (Grade 3 CTCAE) are rare and include bronchopleural fistula, tumor seeding or nerve injury (diaphragmatic or intercostal nerves) [46]. In the largest published series, with 566 patients and 1037 lung metastases [25], two patients died within the 30 days post-procedure, including one with decompensated cardiorespiratory function and one with cerebral stroke. These results are comparable to those found in the surgical series. A multicentric prospective study based on 532 patients and 1050 lung resections, reported that a first episode of lung surgery for lung metastases of colorectal cancer was associated with a very low mortality (<1%) and a postoperative morbidity rate of 16% [47]. This complication rate varies with the surgical technique used, and even if indications for video-assisted thoracic surgery (VATS) have increased, thoracotomy remains the most frequently used approach and wedge resection has become the reference technique.

On the other hand, it is known that any major pulmonary resection is associated with a high risk of postoperative morbidity (between 15 and 47%) and mortality (1 and 12%) [48]. After surgery, 100% of patients require a chest drain and 3% need it for more than 7 days due to air leakage. In addition, one study showed that nearly 25% of patients who undergo thoracic surgery, including thoracotomy and VATS, can develop chronic post-surgical pain, and 33% of them have a neuropathic component [49]. This pain is accompanied by an altered quality of life.

The toxicity profile of SABR is also acceptable, with no peri-procedural mortality. The most common and serious respiratory adverse event (grade 3 CTCAE) is pneumonitis, which affects 3 to 5% of patients in the literature [29]. It is noteworthy that a decrease in lung function after SABR is delayed. A cohort of 127 patients showed a significant decrease in pulmonary function tests (PFT) 12 months after stereotactic radiotherapy (forced expiratory volume in 1 s (FEV1) (−4.1%; *p* = 0.01), corrected diffusing capacity for carbon monoxide (−5.2%; *p* = 0.027), forced vital capacity (−5.7%; *p* = 0.004), and total lung capacity (−3.6%; *p* = 0.039). Declines in FEV1 (−7.6%; *p* = 0.001) and forced vital capacity (−8.9%; *p* = 0.001) persisted at 24 months [50].

Thermal ablation does not significantly change PFT after the procedure [51,52]. Then it appears to be an essential tool for the challenging management of new pulmonary metastases, distant from the ones already treated, because of its good tolerance, parenchymal sparing and high repeatability when needed.

#### 4.3.4. Overall

Although a review of the literature shows a significant increase in the survival of patients with metastatic colorectal cancer, lung ablation or surgical resection is aimed at only a small number of patients. Indeed, the majority of patients with lung metastases have a locally advanced, unresectable primary tumor lesion or other extra-thoracic metastatic lesions, which contraindicates any surgical or percutaneous procedure. The role of thermal ablation in the management of pulmonary metastases of colorectal cancer, like that of surgery and stereotactic radiotherapy, is not clearly defined by the learned societies, probably due to the lack of scientific data with a high level of evidence.

Surgery and thermal ablation do not seem different in terms of technical success, effect on survival or morbidity/mortality. Percutaneous thermal ablation has the advantage of sparing the pulmonary parenchyma, even in the case of repeat procedures. These two techniques can be used in combination. Stereotactic ablative radiotherapy, in the specific case of metastases of colorectal cancer, appears to be less effective than the other two techniques, but is attractive because of its non-invasive nature and the low complication rate.

Finally, the decision will depend on the MDT and will be based on local experience, tumor characteristics and patient preference.

## 5. Conclusions

Image-guided thermal ablation is a reliable and safe technique for the management of colorectal cancer lung metastases. With a careful selection of patients to be treated, it can prolong survival and reduce the need for chemotherapy. Patients for whom percutaneous treatment may have a significant benefit are patients with the following characteristics: <3 pulmonary metastases, nodules diameter <2 cm and for which “R0” treatment is feasible, absence of extra-pulmonary metastatic disease and pulmonary-free disease interval >12 months.

## Figures and Tables

**Table 2:** Adverse events after lung thermal ablation in the 12 previously analyzed studies.

Authors	Pneumothorax	Drain	Pleural Effusion	Fistula	Hemorrage	Pneumonia
T.D. Yan [30]	29%	16%	7%	-	9%	-
K. Yamakado [36]	22%	13%	1%	-	-	-
T.C. Chua [37]	-	33%	-	-	-	-
A. Gillams [38]	-	-	-	-	-	-
T. de Baere [25]	67%	39%			0.2%	
J. Ferguson [31]	54%	19%	1%	1%	1%	-
Y. Matsui [39]	-	-	-	-	-	-
I. Kurilova [32]	58%	38%	-	-	-	2%
M. Fonck [27]	72%	47%	2%	-	-	1%
J. Zhong [40]	60%	30%	3%	3%	-	-
M.R. Calslstrom [26]	-	26%	-	0%	-	-
T. Hasegawa [23]	-	20%	1%	-	1%	-
